# Tapering and Peaking Maximal Strength for Powerlifting Performance: A Review

**DOI:** 10.3390/sports8090125

**Published:** 2020-09-09

**Authors:** S. Kyle Travis, Iñigo Mujika, Jeremy A. Gentles, Michael H. Stone, Caleb D. Bazyler

**Affiliations:** 1 Center of Excellence for Sport Science and Coach Education, Department of Sport, Exercise, Recreation, and Kinesiology, East Tennessee State University, Johnson City, TN 37614, USA; gentlesj@mail.etsu.edu (J.A.G.); stonem@etsu.edu (M.H.S.); bazyler@etsu.edu (C.D.B.); 2 Department of Physiology, Faculty of Medicine and Nursing, University of the Basque Country, 48940 Leioa, Basque Country; inigo.mujika@inigomujika.com; 3 Exercise Science Laboratory, School of Kinesiology, Faculty of Medicine, Universidad Finis Terrae, Santiago 7501015, Chile

**Keywords:** back squat, bench press, deadlift, recovery, periodization, programming

## Abstract

Prior to major competitions, athletes often use a peaking protocol such as tapering or training cessation to improve performance. The majority of the current literature has focused on endurance-based sports such as swimming, cycling, and running to better understand how and when to taper or use training cessation to achieve the desired performance outcome. However, evidence regarding peaking protocols for strength and power athletes is lacking. Current limitations for peaking maximal strength is that many studies do not provide sufficient details for practitioners to use. Thus, when working with athletes such as powerlifters, weightlifters, throwers, and strongman competitors, practitioners must use trial and error to determine the best means for peaking rather than using an evidence-based protocol. More specifically, determining how to peak maximal strength using data derived from strength and power athletes has not been established. While powerlifting training (i.e., back squat, bench press, deadlift) is used by strength and power athletes up until the final days prior to a competition, understanding how to peak maximal strength relative to powerlifting performance is still unclear. Thus, the purpose of this study was to review the literature on tapering and training cessation practices relative to peaking powerlifting performance.

## 1. Introduction

Throughout recorded history, people have performed feats of strength that have left both spectators and athletes alike astonished. As the popularity of strength and power sports such as powerlifting, weightlifting, throwing, and strongman has increased, so have research efforts addressing these sports. Strength is an important fitness characteristic for strength and power sports, particularly powerlifting, and can be defined as the ability to produce maximal force irrespective of the duration of time it takes to achieve a given force output. In competition, powerlifters attempt one-repetition-maximum (1 RM) loads for the three “power lifts”: back squat, bench press, and deadlift. Each lift is contested under strict judging conditions and the maximum loads successfully lifted for each competition lift are summed together for a powerlifting total. Given the focus on strength and the limited number of movements a powerlifter performs in a competition, the primary training adaptation desired for powerlifting is to improve maximal force output in all three competitive lifts. Force production is one of three biomotor abilities (i.e., strength, speed, endurance) used to classify physical skills and has been suggested to be the most important skill to improve sporting tasks [1]. Therefore, strength and power athletes outside of powerlifting often incorporate power lifts in their normal training (e.g., weightlifters back squatting; throwers bench pressing; strongman competitors deadlifting), and in preparation for competition, to improve or maintain sporting tasks. However, powerlifters train with high specificity and do not typically incorporate movements derived from other strength and power sports (e.g., clean-and-jerk; discuss throw at various loads; truck pull) [2]. To improve upper- and lower-body force production, powerlifters often use rigorous training routines with high specificity over several weeks or months leading to a major competition in hopes of performing at their highest level on competition day.

Scientific studies aimed at improving maximal strength often use short-term periodized programs (i.e., 1–4 months) to plan and implement training rather than long-term training programs (i.e., 1 year) [3]. In sport science, long-term training studies are often cut short due to limitations such as athlete availability, coach cooperation, and conflicting holiday and competition schedules. Training for powerlifters typically includes some variation of a periodized training plan or a series of short-term periodized programs (e.g., using three distinct training phases over 12 weeks) with the goal of improving 1 RM performance on competition day [4,5,6]. In a survey that included 32 elite national British powerlifters, nearly all the athletes stated that variations of periodized training models were used to organize training over a competition year [5]. To date, literature addressing the training of powerlifters has included short-term periodized plans implementing training principles from the traditional periodization model (often erroneously referred to as “linear periodization” [7]), various forms of daily undulating periodization (more appropriately classified as Daily Undulating Programming [DUP]), and block periodization [6,8,9,10,11,12]. In the absence of investigations on long-term training programs, short-term periodized programs can inform the best training practices for maximal strength.

Several studies have addressed maximal strength adaptations relative to powerlifting using both competitive powerlifters (i.e., those who compete in sanctioned competitions) and non-competitive powerlifters (i.e., those who train with power lifts regularly and meet a specific relative load-to-body mass lifting ratio, but do not compete in sanctioned competitions) [6,8,10,13,14]. Short-term periodized programs over 6–10 weeks in duration with competitive and non-competitive powerlifters have been shown to elicit powerlifting performance improvements ranging between 2–11% [6,8,10,13,14]. Unfortunately, most studies only attribute performance changes to the effectiveness of the overall program being implemented and do not address pre-competition or pre-testing practices during the final week(s). However, it is possible that the training performed during the final week(s) and days of training is what promotes or hinders performance outcomes [15].

Sport scientists, coaches, and athletes often reduce training by incorporating a taper prior to competitions to manage and mitigate fatigue with the aim of peaking specific fitness characteristics [1,4,16]. The taper has been defined by Mujika and Padilla as “a progressive nonlinear reduction of the training load during a variable period of time, in an attempt to reduce the physiological and psychological stress of daily training and optimize sports performance” [15,17]. A reduction in training load is typically achieved by using the following taper models: a linear fashion (i.e., linear taper), gradually or rapidly in a systematic, exponential fashion (i.e., slow or fast exponential taper), or by a sudden, constant amount (i.e., step taper) [15]. More specifically, as described by Mujika [18], a linear taper implies that a higher total training load is used, compared to an exponential taper, followed by a systematic linear reduction in training load (e.g., a 15% reduction in training load each week for 4 weeks). Additionally, exponential tapers can have a slow or fast time constant decay with the slow decay being similar to a linear taper regarding higher total training loads, yet the reductions are exponentially reduced (e.g., a 60% training load reduction followed by a 40% training load reduction) [18]. Lastly, the step taper can be considered a reduced training procedure where the training load is suddenly reduced by a constant amount (e.g., 50%), which is often associated with maintaining performance but may also improve performance [18].

Regardless of the tapering model selected, the taper is often regarded as a key phase or portion of any given training regimen [19]. Additionally, following a taper or in place of a taper, training cessation may be implemented. Training cessation can be defined as planned days of complete rest where all sporting activities cease whilst continuing everyday activities [20]. Training cessation has been shown to be most effective over ≤7 days (i.e., short-term training cessation) to promote recovery, resulting in maintained or improve performance [20,21]. However, if the training stimuli are removed for prolonged periods of time (>14 days), this may result in detraining [21]. Unfortunately, most resistance training studies do not state if, when, or how a taper or training cessation is implemented. Many powerlifters as well as other strength and power athletes, only compete 1–3 times per year. Thus, understanding how and when to structure a taper or training cessation for the power lifts is vital to achieve optimal performance [4].

Tapering and training cessation for strength and power athletes are becoming prevalent topics in sport science research. The topics of tapering and training cessation have been documented in the literature for endurance performance [21,22,23,24,25,26,27,28], maximal power performance [29,30,31], and more recently individual and team sport performances [32,33]. However, there is very limited evidence regarding tapering for maximal strength [34], particularly as it relates to the power lifts. This is important considering that most strength and power athletes implement the power lifts to some degree in their normal and pre-competition training regimens to improve or maintain maximal strength and, in turn, competition outcomes. Additionally, the efficacy of using training cessation to improve maximal strength has also been questioned. Thus, the purpose of this study is to review the literature on tapering and training cessation practices for powerlifting performance.

## 2. Materials and Methods

### 2.1. Search Strategy

This review was conducted according to the Preferred Reporting Items for Systematic Reviews and Meta-Analyses (PRISMA) guidelines. A literature search was conducted from November 2019 to January 2020 using the following databases: Google Scholar, PubMed, ScienceDirect, and Open Access Theses and Dissertations. There were no limitations regarding publication date. The search and retrieval of manuscripts were conducted by using the search terms “powerlift/ing,” “back squat,” “bench press,” “deadlift,” AND “taper/ing” OR “peak/ing” OR “cessation.” The search results were downloaded and filtered in Zotero software (version 5.0.77 October 2019). Original research articles published in peer-reviewed journals, as well as unpublished materials that included all data in detail, were considered for review. A secondary search was performed by screening the reference lists of all articles obtained that were not identified electronically and a forward citation tracking (using Google Scholar) of studies was conducted.

### 2.2. Inclusion and Exclusion Criteria

**Figure 1:**
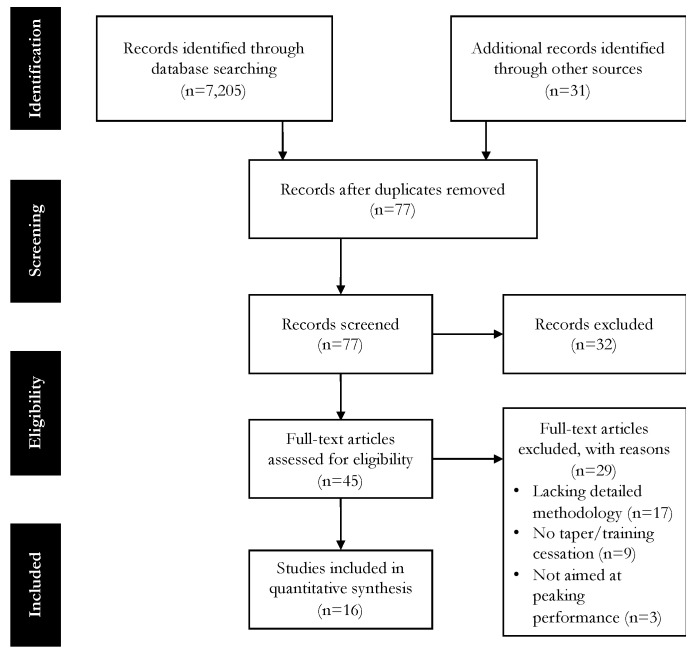
Preferred Reporting Items for Systematic Reviews and Meta-Analyses (PRISMA) flow diagram.

To warrant inclusion and relevance in the current analysis, potential studies were required to meet the following criteria: (1) involved competitive individual strength-power sport athletes (i.e., powerlifters, weightlifters, throwers, strongman competitors) or non-competitive strength-trained subjects/recreationally strength-trained subjects with homogenous lifting criteria (e.g., non-competitive powerlifters who can back squat ≥ 150%, bench press ≥ 125%, and deadlift ≥ 150% body mass); (2) incorporated a peaking protocol (i.e., defined by using a taper, reduced training period, or training cessation); or (3) the peaking protocol was performed prior to competitions, simulated competitions, or 1 RM testing for the back squat, bench press, deadlift, or maximal effort laboratory test(s) related to powerlifting performance/maximal strength (e.g., isometric back squat, isometric bench press). A total of 7205 articles was identified electronically. Duplicates were discarded by placing titles in alphabetical order in Zotero. If the article’s title or abstract was not related to strength-power athletes or strength-trained subjects preparing for 1 RM or maximal effort testing, lacked methodology details, had no implications for competition powerlifting performances, or was not written in English, the article was discarded and excluded from the study. A manual search was performed from the reference lists of all articles considered and cross-referenced through Google Scholar (Figure 1).

After the initial identification of articles, reference screening, and removal of duplicates, 77 articles were further screened for inclusion. After screening the titles and abstracts, 45 articles appeared to be eligible to be included in the review. The full text of the 45 articles was further assessed for eligibility and 29 articles were excluded due to lacking details regarding methodology (*n* = 17), not providing specifics about a taper or training cessation being used (*n* = 9), or not being aimed at achieving maximal strength for powerlifting performance (*n* = 3). There were two surveys involving powerlifters [35,36] and one survey involving strongman competitors [37] that were included in the review that did not provide performance outcomes. However, inclusion of these studies can be justified considering they provide the most detailed account of tapering to date for powerlifting performance. Excluding the large strongman tapering practices survey [37], studies involved sample sizes ranging from 5 to 44 subjects (Table 1).

### 2.3. Quality of Studies

To reduce the risk of study selection bias, the first author and an uninvolved independent collaborator conducted the search for studies using the search terms provided within the specified databases. A third party screened the search results of all studies included. Any disagreements were discussed, and the third party made the final decision. Study quality was assessed using a modified version of the Tool for the Assessment of Study Quality and reporting in Exercise (TESTEX) scale [38]. The original scale ranges from 0 to 15, and higher scores represent the higher methodological quality of the studies. However, this scale was originally created for “chronic studies” and, therefore, a modified version of the TESTEX was used for “acute studies” with a scale ranging from 0 to 7 [38,39]. Study quality was scored based on the following categories: (1) subject eligibility specified (1 point); (2) cohort similar at baseline (1 point); (3) outcome measures assessed (2 points); (4) statistical reporting (1 point); and (5) training intensity and volume changes specified (2 points). The final 16 articles were evaluated for quality resulting in scores of 3-7, and all relevant data were extracted from each article and categorized by tapering effects on powerlifting performance outcomes, training cessation effects on powerlifting performance outcomes, and tapering studies only involving samples of powerlifters.

### 2.4. Data Analysis

The first author read all of the included studies to gain familiarity and then subsequently re-read and extracted relevant data. The extracted data were used to facilitate analysis and presentation which included: (1) author and year, (2) sample size and demographics, (3) taper model (e.g., step taper, exponential taper, linear taper), (4) taper duration (i.e., expressed in days or weeks), (5) intensity (i.e., percentage of 1 RM) and (6) volume (i.e., set × repetitions × training load) manipulations, (7) competition based performance outcomes (expressed in percentage change and absolute terms (g)), and (8) laboratory-based performance outcomes (expressed in percent change and absolute terms). Any corresponding statistical reporting was retrieved and included where applicable.

## 3. Results

Seven studies involved tapering for back squat, bench press, or isometric bench press performance with powerlifters (number of studies: *n* = 2; total number of subjects: *n* = 33), weightlifters (*n* = 2; *n* = 36), throwers (*n* = 1; *n* = 9), and strength-trained subjects (*n* = 2; *n* = 25) (Table 2). Within each study, it appeared that only exponential tapers (*n* = 7) and step tapers (*n* = 5) were used for various cohorts and lasted 7, 14, or 28 days. Intensities were either maintained, reduced between 8.5–25.0%, or increased by 5.9% but all studies reduced the volume ranging between 31.6–71.9%. However, the manuscripts by Godawa et al. [40] state that “weeks 9 and 10 was the tapering period in which both volume and intensity decreased,” but based on the figures provided, it appears that volume was increased during weeks 9 and 10 whereas only intensity decreased by −25.0% relative to pre-taper training. All noted back squat (1.7–9.5%, 2.0–14.8 kg) and bench press (1.4–6.4%, 1.3–8.1 kg) performances improved, but isometric bench press peak force scaled to body mass did not change by a notable margin (0–2.7%; 0.0–0.5 N).

Nine studies implemented training cessation, which included track and field athletes (*n* = 1; *n* = 41), powerlifters (*n* = 4; *n* = 30), and American football players (*n* = 1; *n* = 8), strongman competitors (*n* = 1; *n* = 423), and strength-trained subjects (*n* = 3; *n* = 47) (Table 3 and Table 4). Training cessation was typically implemented between 2–14 days. However, the majority of performance improvements were only noted with short-term training cessation (≤7 days) for back squat (1.7–4.9%, 2.0–5.5 kg), bench press (1.4–4.9%, 1.3–5.5 kg), as well as isometric bench press peak force allometrically scaled to body mass (1.0–1.5%) and rate of force development (9.5%). Interestingly, several performance decrements were noted only for isometric bench press with short-term training cessation. Isometric peak force output diminished at 2 days cessation (effect size (ES): −0.13) and at 3 days cessation (ES: −0.06, −0.11). Isometric peak force scaled to body mass diminished at 2 days (ES: −0.11), 3 days (ES: −0.03), and 5 days (ES: −0.03) of cessation. Isometric rate of force development also decreased at 3.5 days of cessation (−8.0%). Training cessation over 14 days appeared to decrease both back squat and bench press 1 RM performance (−0.9% (ES: 0.05)) and −1.7% (ES: 0.12), respectively).

There were 6 studies that only involved powerlifting cohorts (Table 5). Powerlifters competed at the local club level (*n* = 15), collegiate level (*n* = 23), national level (*n* = 15), international level (*n* = 11), or were non-competitive (*n* = 5). Exponential (*n* = 4; *n* = 35) and step tapers (*n* = 3; *n* = 29) were used similarly, while only one study incorporated a linear taper with a small sample of collegiate powerlifters (*n* = 5). These tapers varied in intensity and volume, similar to what was previously mentioned, but with volume reductions ranging between 31.6–67.0% spanning 7–28 days. No performance decrements were reported. Improvements were noted for back squat (2.3–5.9%, 3.6–14.5 kg), bench press (1.8–6.4%, 2.3–8.1 kg), deadlift (3.8–4.8%, 8.6–9.1 kg), powerlifting total (3.2–4.4%, 14.1–27.7 kg), and Wilks Score (2.8–4.9%, 11.0–16.0 au).

## 4. Discussion

The purpose of this study was to review the literature on tapering and training cessation practices for powerlifting performance. Due to the paucity of literature, the studies summarized in this review considered not only powerlifters, but similar strength and power athletes (i.e., weightlifters, throwers, strongman competitors, American football players) and strength-trained subjects who often use a back squat, bench press, and deadlift in their normal training routines. By only including studies from homogenous samples, we negated the dissolution of drawing inferences from samples that do not align with powerlifters (e.g., soccer players, basketball players). Thus, implications may be more accurately applied when sport scientists and coaches incorporate these tapering and peaking strategies with powerlifters or to enhance powerlifting performance. Unfortunately, the majority of tapering and training cessation studies only included data on back squat and bench press performances, whereas deadlift and powerlifting total performances as well as Wilks Score changes were not as frequently reported. Additionally, the studies highlighted in this review incorporated male and female athletes ranging from local to international level competition as well as well-trained strength-trained males. Based on our findings, tapering and short-term training cessation both appear to be effective for improving powerlifting performance. The tapering protocols included in this review agree with the current literature and also provide novel insights into some unconventional tapering practices.

While the four classic methods of tapering have been previously defined and were used to highlight the tapers implemented in the current review, it is important to note that in some cases, linear and exponential tapers have been previously grouped together as “progressive tapers” [15,22]. Although the linear and exponential taper models indeed use a progressive reduction in the training load over time, it was paramount to describe the tapers relative to how the progressive reductions were implemented (i.e., linearly, exponentially). In a meta-analysis by Bosquet et al. [22], progressive tapers were associated with greater performance improvement compared to step tapers with endurance athletes. Additionally, the authors suggested that step tapers were suitable for maintaining performance. However, for maximal strength performance, it appears that step tapers may improve maximal strength to the same degree or greater compared to other tapering models. Regardless, tapering with an exponential model or step model appears to be preferred in order to improve powerlifting performance. More importantly, it may be possible that the volume reduction is what determines the performance outcome more than the taper model implemented.

For endurance sports, a training volume reduction of 40–70% over a 2–3 week period is recommended to significantly improve performance [15,22,28]. Similar recommendations have been provided for maximal strength [34]; however, the volume reduction recommendation was 30–70% and a taper duration of up to 4 weeks. In the current analysis, it appears that small-to-moderate volume reductions (~30 to ≤ 50%) seem to elicit greater performance outcomes compared to larger reductions (>50 to ≤ 70%) for back squat and bench press performance, particularly over a 2-week period (Table 2). The smallest volume reductions in the current analysis were 32% and 37%, yielding a ~4% improvement on back squat and bench press performance with powerlifters and weightlifters [41,42]. Using a 2-week step taper with national level throwers, Kyriazis et al. [43] reduced intensity and volume (exact specifications were not detailed), which improved back squat 1RM by 6% and throwing performance by 5%. In another study with throwers, Zaras et al. [44] decreased the volume by 25–40% over 2 weeks, resulting in improved throwing performances to a similar degree (5–6%). However, with only a 25% volume reduction, the performance change was smaller relative to the 40% volume reduction. Therefore, powerlifters may want to avoid volume reductions that are too small (≤25%) and safely implement a reduction of at least 30–35% to elicit performance improvements.

Furthermore, evidence suggests that the volume reduction can be as high as 90% with endurance athletes [45]. However, these large volume reductions (≥70%) may not be warranted when attempting to peak maximal strength to prevent maladaptation and detraining [17,18]. For example, with a national level female weightlifter, a volume reduction of >70% over a 3-week taper resulted in decreased weightlifting competition performance (−2%) and laboratory performance decrements (loaded and unloaded jumps, isometric mid-thigh pull) [46]. Likewise, Pritchard et al. [47] showed no change in isometric bench press performance after a volume reduction of >70% over a 7-day taper. Interestingly, the opposing group did not exceed a reduction of 70% which resulted in a positive performance change (3%). Pritchard et al. [47] attempted to reduce both groups’ training by 70% with the primary aim of manipulating intensity by 5% and −10%. However, larger volume reductions may be needed and necessary after a planned overreach (i.e., a mild increase in the overall training stimuli to elicit a performance improvement [48]) prior to a short taper (7 days). For example, over a 3-week peaking protocol implemented by Williams [42], volume was reduced by 32% relative to normal training from week 1 and 1RM bench press performance improved by 4%. However, during week 2, Williams [42] implemented a planned overreach week by increasing volume by 107% prior to the taper. During the 1-week taper on week 3, volume was reduced by 67% relative to the planned overreach volume, and bench press performance improved by 6%. The aforementioned national level female weightlifter was prescribed a planned overreach week prior to a 3-week taper [46], whereas the strength-trained males tapered from normal training [47]. Therefore, it is possible that a planned overreach followed by a large volume reduction of <70% can aid in rebounding performance during a short taper (7 days). Studies with reports of increased or maintained intensity, with large volume reductions, have also observed decreased muscle size relative to baseline or pre-taper values. For example, decreases in vastus lateralis cross-sectional areas have been observed following 3 weeks of tapering in national level weightlifters, possibly due to insufficient training volume [46,49]. Thus, a short taper (7 days), or a slight increase in volume of ≤10% over 2–3 weeks may afford athletes with a small but meaningful fitness improvement leading into a competition, as demonstrated by Godawa et al. [40] In the current analysis, it appears that intensity manipulations may not dictate performance changes to the same degree as volume manipulations.

The general recommendation for tapering is to increase or maintain intensity [15,22,28,34]. However, to peak maximal strength for powerlifting performance, it appears that intensity can be increased during the taper, but during the final days, intensity is either maintained or decreased to promote recovery. For powerlifting, increasing the intensity can only be done by a small margin (≤15%) considering that normal training is typically ≥85% 1RM. Reducing volumes to a large extent and increasing high-intensity work could lead to negative performance outcomes, or could inhibit the athlete from improving performance [50] as demonstrated by Pritchard et al. [47] Although some aspects of powerlifting performance improved with increased intensity [51,52], maintaining intensity may be a safer option when constructing a taper for maximal strength. For example, studies by Häkkinen et al. [53] and Seppänen [54] showed that powerlifters and strength-trained individuals both improved performance by maintaining training intensity at ~85%, while reducing volume by approximately 50–54% over 1–2 weeks. The national powerlifters of Croatia reported performing a similar taper [36], but the duration typically spanned 18 days. The tapering parameters reported by the New Zealand national powerlifting team were similar in duration (17 days) [35], but suggested that intensity is typically reduced by 5%. Studies that maintained intensity appeared to produce performance improvements of 1–6% [42,54], whereas those that decreased intensity appeared to produce performance improvements of 2–10% [40,41,43,47,55]. The studies that reported increased intensity [47,51,52] elicited either no performance change or an unspecified overall performance improvement. However, for the positive performance outcomes mentioned for collegiate powerlifters [51,52], intensity was increased up to the last training session and then decreased by 4% for the final training session followed by training cessation leading into a competition. Drastic intensity reductions were also noted by Godawa et al. [40] during the final training week leading into a competition with collegiate powerlifters. The tapering intervention implemented by Godawa et al. [40] induced positive performance changes for all powerlifting performance ranging from 2 to 6% by tapering intensity exponentially by 25%, while slightly increasing the volume over a 2-week period [40]. Increasing volume is typically seen through intensified training or planned overreaching and not tapering [56,57]. Thus, this unconventional method of tapering needs further investigation.

Training cessation can be effective in terms of maintaining or improving performance if implemented appropriately over a proper duration [20,21]. It has been proposed that maximal strength adaptations can be maintained for up to 30 ± 5 days if training is completely removed due to a residual training effect [58]. However, synthesized experimental evidence has shown training cessation >7 days results in decreased maximal strength performance ranging from 1–4% [17,21,59,60]. A meta-analysis by Bosquet et al. [21] indicated that maximal force declines at similar rates with a cessation period of < 7 and 7–14 days, but begins to diminish rapidly ≥15 days. Hortobàgyi et al. [59] showed that after 14 days of complete rest, powerlifters and American football players decreased their maximal strength for back squat and bench press, albeit not to a statistically significant degree. This performance decline is likely attributed to a lack of stimuli across the cessation period. Studies by Gibala, MacDougall, and Sale, [61] and Izquierdo et al. [62] provide additional evidence suggesting that neuromuscular adaptions begin to diminish at 10 days of training cessation and at 28 days significant reductions are noticed in back squat (−6%) and bench press (−9%) 1RM. When assessing well-trained athletes, as highlighted in this review, their eccentric force and sport-specific power, and recently acquired strength, may decline significantly over ~30 days [63]. Thus, training cessation should not be implemented to the extent that detraining occurs [17].

Conversely, training cessation durations that are too short (e.g., ≤1 day) may also disallow optimum biological and psychological restoration to take place. For instance, Weiss et al. [64] have shown that within 2, 3, 4, and 5 days of training cessation, bench press 1RM can improve, but isometric bench press measurements may decrease. Interestingly, days 2 and 3 of training cessation were the only days where isometric peak force diminished similarly but provided the highest bench press 1RM outcomes. Pritchard et al. [20] recently corroborated the findings by Weiss et al. [64] showing that 3.5 days of training cessation diminished the isometric bench press rate of force development when compared to 5.5 days, although no overall effects on strength measures were observed. However, Weiss et al. [64] also showed that 1RM bench press performance improved to the greatest extent with as little as 2 days of complete rest. Similarly, over 2, 5, and 7 days of training cessation, Anderson and Cattanach [65] observed a 5% average improvement in 1RM back squat and bench press. Therefore, 2–7 days of training cessation appear to be sufficient to maintain, or possibly improve, powerlifting performance. Reports from strongman competitors appear to agree with this duration for back squat and bench press, but deadlift training may cease over a slightly longer period of time [37]. For national- and international-level powerlifters, longer durations of training cessation specifically for the deadlift appear to be common practice [35,36]. The deadlift may be completely removed and not trained for 1–2 weeks leading into a competition [35,36,37]. While recent studies suggest that recovery times are similar between back squat, bench press, and deadlift [66,67], the actual tapering practices of high-level strength athletes disagree [35,36,37].

Considering the lack of tapering studies performed on powerlifters, Table 5 provides evidence from the available literature implementing various tapers in an attempt to peak powerlifting performance with powerlifters. Häkkinen et al. [53] investigated the effectiveness of reducing the volume by 50% with competitive national Finnish powerlifters. It was suggested that performance can be brought to a peak when volume is cut in half over 1 week. While this study implemented a 1-week step-wise reduction in volume of 50%, Grgic and Mikulic, [36] and Pritchard et. al. [35] reported similar reductions (51–59%) over 17–18 days with national and international level powerlifters, although no performance data were included. Williams [42] also showed that a 1-week step-wise taper with an average reduction of 50% was sufficient to improve bench press 1RM by an average of 5% with US powerlifters. Conversely, Godawa et al. [40] tapered intensity by 10% during the first week of the taper followed by 15% during the final week of the taper while slightly increasing volume, and still observed a positive performance outcome for all powerlifting performances ranging between 2–6%. While Godawa et al. [40] noted statistically significant changes for back squat, deadlift, powerlifting total, and Wilks Score improvements, there were no significant improvements on bench press. Godawa et al. [40] did report, however, smaller bench press 1RM performance improvements (2% for equipped lifters, 2% for classic raw lifters) compared to Williams [42] and a 5% average improvement. Nevertheless, these performance discrepancies may be attributed to the sex, age, or level of competition between lifters in each study.

Nevertheless, maintaining or reducing intensity for powerlifters may allow neuromuscular recovery and adaptation to occur. For example, national level Finnish powerlifters were able to improve both maximal neural activation, as determined by electromyographic activity for the quadriceps (excluding the intermedius muscle), and maximal force per unit of quadriceps femoris muscle cross-sectional area at the end of the 1-week taper [53]. Häkkinen et al. [53] indicated that maximal strength performance in highly trained strength athletes may be brought to peak levels after a short duration of reduced volume due to neuromuscular recovery. Reducing the training volume by half to facilitate recovery while maintaining training intensity (e.g., ≥85% 1RM) may be enough to prevent detraining and peak force production. Interestingly, based on isometric bench press performances observed by Weiss et al. [64], 4 days of cessation appears to elicit the highest degree of force production improvements, which may be attributed to additional neuromuscular recovery, whereas 2 days of cessation elicited the highest 1RM improvement. The tapering intervention implemented by Andre, Askow et al. [51,52] provides additional evidence that powerlifting performance improvements take place following 4 days of training cessation. After increasing intensity for 3 weeks by 10%, during the final week of a 28-day linear taper, intensity was reduced by 4% for the last training session, followed by 4 days of complete rest prior to the competition. All powerlifters (*n* = 5) reportedly improved competition performances and set 7 state records on the day of the competition [51,52]. While the tapering intervention appeared to be successful, the performance improvements could also be attributed to the training adjustments implemented during the final week of the taper.

## 5. Conclusions

Based on the evidence reviewed, strength athletes tapering to improve powerlifting performance should (1) reduce training volume by approximately 30–70%, (2) maintain training intensity ≥85% 1RM or reduce training intensity while (3) using either an exponential or step taper model to manipulate the distribution of work over a 1–2 week period followed by (4) a short-term training cessation spanning 2–7 days. Our guidelines agree with findings by Pritchard et al. [34] with the exceptions that (1) optimal taper duration may only be 2 weeks and (2) intensity can also be decreased, particularly during the week of competition, to improve performance.

The effectiveness of the taper may be determined by the distribution of work followed by complete rest leading up to a competition. However, some athletes may need modifications outside the recommended ranges to achieve their desired performance outcomes. Future studies investigating tapering for maximal strength should include detailed information regarding the construction and implementation of the taper. Studies should also compare the effectiveness of tapering models (e.g., step taper vs. exponential taper) and compare the effects of tapering versus only using training cessation (e.g., 1-week step taper vs. 1-week training cessation). Additionally, a limitation of this study is that we cannot account for the potential use of anabolic steroids nor other performance-enhancing drugs that are commonly used in strength and power sports [68,69,70,71]. It is possible that athletes who use such substances require different recovery periods, and therefore, different tapering parameters prior to competition. Lastly, we cannot account for any activities that subjects were involved in outside the strength training interventions. This review provides an evidence-based approach for powerlifters aiming to peak for competition.

## Figures and Tables

**Table 1:** Reported Demographics and Study Quality.

Author and Year	Competition Level/Status	Athlete Type/Sample Size	Sex/Sample Size	Age (years)	Body Mass (kg)	Height (cm)	Quality Score (%) *
Häkkinen et al. 1991	NAT/NC	PL (*n* = 10)	M (*n* = 10)	29.2 ± 5.8	75.0 ± 15.0	-	6 (86%)
Anderson and Cattanach 1993	D1	TF (*n* = 41)	M (*n* = 22); F (*n* = 19)	-	-	-	5 (71%)
Hartman et al. 2004	NAT	WL (*n* = 7)	M (*n* = 7)	19.7 ± 1.6	94.0 ± 21.1	-	3 (43%)
Weiss et al. 2004	NC	ST (*n* = 25)	M (*n* = 25)	24.2 ± 3.8	89.0 ± 0.9	-	6 (86%)
Hortobáygi et al. 2008	C/D1	PL (*n* = 4); AFB (*n* = 8)	M (*n* = 12)	24.4 ± 0.7	88.6 ± 3.6	181.1 ± 10.1	7 (100%)
Kyriazis et al. 2009	NAT	TH (*n* = 9)	M (*n* = 9)	26.0 ± 4.0	113.3 ± 9.0 ^†^	188.4 ± 6.0	3 (43%)
Godawa et al. 2012	C	PL (*n* = 10)	M (*n* = 8); F (*n* = 2)	21.5 ± 3.5	80.7 ± 38.5	175.3 ± 25.1	6 (86%)
		EQ-PL (*n* = 8)	M (*n* = 6); F (*n* = 2)	22.0 ± 5.7	94.1 ± 44.6	176.6 ± 16.2	
Andre, Askow et al. 2016	C/Jr.	PL (*n* = 5)	M (*n* = 5)	21.0 ± 4.2	111.3 ± 32.8	179.0 ± 6.0	5 (71%)
Gonzàlez-Badillo et al. 2016	NAT/Jr.	WL (*n* = 12; LIG)	M (*n* = 29)	17.1 ± 1.7	73.7 ± 5.5	168.0 ± 4.1	4 (57%)
		WL (*n* = 9; MIG)		16.9 ± 1.7	74.0 ± 3.9	167.0 ± 4.0	
		WL (*n* = 8; HIG)		17.5 ± 1.9	72.0 ± 2.3	169.1 ± 3.6	
Pritchard et al. 2016	INT	PL (*n* = 11)	M (*n* = 8); F (*n* = 3)	28.4 ± 7.0	91.0 ± 27.4	-	3 (43%)
Grgic and Mikulic 2017	NAT	PL (*n* = 10)	M (*n* = 6);	29.8 ± 3.8	86.3 ± 16.8	-	3 (43%)
			F (*n* = 4)	28.3 ± 2.2	64.2 ± 9.4	-	
Williams 2017	CL	PL (*n* = 15)	M (*n* = 12); F (*n* = 3)	25.0 ± 6.0	93.0 ± 17.6	175.8 ± 7.9	7 (100%)
Pritchard et al. 2018	NC	ST (*n* = 11)	M (*n* = 11)	21.3 ± 3.3	92.3 ± 17.6	182.0 ± 8.0	7 (100%)
Pritchard et al. 2018	NC	ST (*n* = 8)	M (*n* = 8)	23.8 ± 5.4	79.6 ± 10.2	180.0 ± 6.0	7 (100%)
Seppänen 2018	NC	ST (*n* = 7; Group 1)	M (*n* = 14)	26.1 ± 2.8	84.2 ± 11.2	183.1 ± 5.5	7 (100%)
		ST (*n* = 7; Group 2)		25.6 ± 2.6	81.7 ± 9.4	180.0 ± 3.5	
Winwood et al. 2018	CL/NAT/INT	SM	M (*n* = 353); F (*n* = 101)	33.2 ± 8.0	108.6 ± 27.9	178.1 ± 10.6	4 (57%)

Notes: M = male; F = female; CL = club/local; NAT = national; INT = international; D1 = Division 1; C = collegiate; Jr. = Junior Division; NC = non-competitive; PL = powerlifters; EQ-PL = equipped powerlifters (i.e., lifting suits allowed in competition); WL = weightlifters; TH = throwers; TF = track and field athletes; SM = strongman competitors; AFB = American football players; ST = strength-trained subjects. * = modified TESTEX scale score and percentage in relation to the total. LIG = low intensity group; MIG = moderate-intensity group; HIG = high-intensity group. ^†^ = denotes the average of pre- and post-study measurement.

**Table 2:** Effects of tapering on back squat, bench press, and isometric bench press performance.

Author and Year	Athlete	Sample Size	Taper Model	Duration	Intensity	Volume	BS-1RM	BP-1RM	IBP-PFa
Williams 2017	PL	*n* = 15	Step	7 days	↑↓	↓ 67.0% ^†^/↓ 31.6%	-	↑ 6.4%; 8.1 kg (*p* < 0.05) ^†^/ ↑ 3.7%; 4.8 kg (*p* <0.05)	-
Pritchard et al. 2018	ST	*n* = 11	Step	7 days	↑ 5.9%	↓ 71.9%	-	-	↑↓
	ST	*n* = 11	Step	7 days	↓ 8.5%	↓ 70.0%			↑ 2.7%; 0.5 N
Kyrazis et al. 2009	TH	*n* = 9	Step	14 days	↓	↓	↑ 6.5%; 14.0 kg (*p* < 0.025)	-	-
Seppänen 2018	ST	*n* = 7	Step	14 days	↑↓	↓ 54.0%	↑ 3.4%; 4.3 kg (*p* = 0.003)	↑ 2.0%; 2.0 kg (*p* = 0.099)	-
	ST	*n* = 7	Exponential	14 days	↑↓	↓ 54.0%	↑ 1.7%; 2.0 kg (*p* = 0.04)	↑ 1.4%; 1.3 kg (*p* = 0.076)	
Godawa et al. 2012	PL	*n* = 10	Exponential	14 days	↓ 25%	↑	↑ 2.3%; 3.6 kg	↑ 2.1%; 2.3 kg	-
	EQ-PL	*n* = 8	Exponential	14 days	↓ 25%	↑	↑ 5.9%; 14.5 kg	↑ 1.8%; 2.7 kg	
González-Badillo et al. 2016	WL	*n* = 12	Exponential	14 days	↓	↓ 50%	↑5.3%; 8.2 kg (*p* < 0.01)	-	-
	WL	*n* = 9	Exponential	14 days	↓	↓ 50%	↑ 9.5%; 14.8 kg (*p* < 0.05)		
	WL	*n* = 8	Exponential	14 days	↓	↓ 50%	↑ 6.9; 11.1 kg (*p* < 0.05)		
Hartman et al. 2004	WL	*n* = 7	Exponential	28 days	↓ 15.0%	↓ 37.0%	↑ 3.9%; 7.2 kg	-	-

Notes: BS-1RM = back squat 1-repetition-maximum; BP-1RM; bench press 1-repetition-maximum; IBP-PFa = isometric bench press peak force allometrically scaled to body mass; PL = powerlifters; EQ-PL = equipped powerlifters; WL = weightlifters; TH = throwers; ST = strength-trained subjects; ↓↑ = maintained; ↓ = decreased; ↑ = increased. ^†^ = indicates outcome and result post-overload week (i.e., increased volume-load similar to planned overreaching). Williams conducted a 3-week study where week 1 was normal training, week 2 was an overload week, and week 3 was a taper. Williams compared week 2 to week 3/week 1 to week 3 outcomes. Godawa et al. tapered intensity by −10% during the first 7 days of the taper followed by −15% during the final 7 days of the taper. When combing groups, Godawa et al. showed statistical significance for combined BS-1RM (*p* = 0.02) improvements. González-Badillo et al. stated that volume was reduced by 60% during the first 7 days of the taper followed by 40% during the final 7 days of the taper. For Seppänen’s exponential group, the planned reduction was similar to González-Badillo et al. but the actual reduction resulted in a 54% reduction. Pritchard et al. planned for a 5% increase and a −10% decrease with intensity but resulted in 5.9 and −8.5, respectively.

**Table 3:** Effects of training cessation on back squat, bench press, and isometric bench press performance.

Author and Year	Athlete	Sample Size	Cessation Duration	BS-1RM	BP-1RM	IBP-PF	IBP-PFa	IBP-RFD
Seppänen 2018	ST	*n* = 7	2 days	↑ 3.4%; 4.3 kg (*p* = 0.003)	↑2.0%; 2.0 kg (*p* = 0.099)	-	-	-
	ST	*n* = 7	2 days	↑ 1.7%; 2.0 kg (*p* = 0.04)	↑ 1.4%; 1.3 kg (*p* = 0.076)			
Pritchard et al. 2018	ST	*n* = 8	3.5 days	-	-	-	↑ 1.5%; 0.3 N	↓ 8.0%; 683.3 N·s^−1^
	ST	*n* = 8	5.5 days				↑ 1.0%; 0.2 N	↑ 9.5%; 822.3 N·s^−1^
Andre, Askow et al. 2016	PL	*n* = 5	4 days	↑	↑	-	-	-
Weiss et al. 2004	ST	*n* = 8	2 days	-	↑ (ES = 0.15)	↑ (ES = 0.12)/ ↓ ES = −0.13	↑ (ES = 0.27)/ ↓ ES = −0.11	-
	ST	*n* = 5	3 days		↑ (ES = 0.08)	↓ ES = −0.11/ ↓ ES = −0.06	↑ (ES = 0.10)/ ↓ ES = −0.03	
	ST	*n* = 5	4 days		↑ (ES = 0.03)	↑ (ES = 0.26)/ ↑ ES = 0.02	↑ (ES = 0.30)/ ↑ (ES = 0.03)	
	ST	*n* = 7	5 days		↑ (ES = 0.07)	↑ (ES = 0.07)/ ↑ (ES = 0.00)	↑ (ES = 0.05)/ ↓ ES = −0.03	
Anderson and Cattanach, 1993	TF	*n* = 41	2, 4, or 7 days	↑ 4.9%; 5.5 kg	↑ 4.9%; 5.5 kg	-	-	-
Hortobáygi et al. 2008	PL/AFB	*n* = 4/*n* = 8	14 days	↓ 0.9%; 1.7 kg (*p* < 0.05)	↓ 1.7%; 2.3 kg (*p* < 0.05)	-	-	-

Notes: BS-1RM = back squat 1-repetition-maximum; BP-1RM; bench press 1-repetition-maximum; IBP-PF = isometric bench press peak force; IBP-PFa = isometric bench press peak force allometrically scaled to body mass; IBP-RFD = isometric bench press rate of force development; ST = strength-trained subjects; PL = powerlifters; TF = track and field athletes; AFB = American football players; ES = effect size.↓ = decreased; ↑ = increased. Weiss et al. IBP-PF and IBP-PFa results represent PF at 0.37 m·s^−1^/PF at 1.49 m·s^−1^. Anderson and Cattanach state that the 4.9% (5.5 kg) increase is a grand mean total for both BS-1RM and BP-1RM.

**Table 4:** Training cessation practices extracted from qualitative reports.

Author and Year	Athlete	Sample Size	Cessation Duration
Grgic and Mikulic 2017	PL	*n* = 10	2–4 days
Pritchard et al. 2016	PL	*n* = 11	2–5 days
Winwood et al. 2018	SM	*n* = 250	2–6 days
	SM	*n* = 161	3–10 days (for back squat only)
	SM	*n* = 91	4–8 days (for bench press only) ^†^
	SM	*n* = 171	5–11 days (for deadlift only)

Notes: PL = powerlifters; SM = strongman competitors. ^†^ = bench press cessation duration was statistically shorter (*p* < 0.001) than deadlift cessation. No performance outcome data was provided for qualitative studies.

**Table 5:** Summary of tapering practices used with powerlifters and the effects of tapering on powerlifting performance.

Author and Year	Athlete	Sample Size	Taper Model	Duration	Intensity	Volume	BS-1RM	BP-1RM	DL-1RM	PT	Wilks Score
Häkkinen et al. 1991	PL/NC-PL	*n* = 5/*n* = 5	Step	7 days	↓↑	↓50.0%	↑	-	-	-	-
Williams 2017	PL	*n* = 15	Step	7 days	↑↓	↓ 67.0% ^†^ ↓ 31.6%	-	↑ 6.4%; 8.1 kg (*p* < 0.05) ^†^/ ↑ 3.7%; 4.8 kg (*p* < 0.05)	-	-	-
Godawa et al. 2012	PL	*n* = 10	Exponential	14 days	↓ 25%	↑	↑ 2.3%; 3.6 kg	↑ 2.1%; 2.3 kg	↑ 4.8%; 8.6 kg	↑ 3.2%; 14.1 kg	↑ 4.9%; 16.0 au
	EQ-PL	*n* = 8	Exponential	14 days	↓ 25%	↑	↑ 5.9%; 14.5 kg	↑ 1.8%; 2.7 kg	↑ 3.8%; 9.1 kg	↑ 4.4%; 27.7 kg	↑ 2.8%; 11.0 au
* Pritchard et al. 2016	PL	*n* = 11	Exponential	17 days	↓ 5.0%	↓ 58.9%	-	-	-	-	-
* Grgic and Mikulic 2017	PL	*n* = 10	Exponential Step	18 days	↓↑/↑ 5.0%	↓ 50.5%	-	-	-	-	-
Andre, Askow et al. 2017	PL	*n* = 5	Linear	28 days	↑ 10.0%/↓ 4%	↓ 58.7%	↑	↑	↑	↑	↑

Notes: BS-1RM = back squat 1-repetition-maximum; BP-1RM; bench press 1-repetition-maximum; DL-1RM = deadlift 1-repetition-maximum; PT = powerlifting total; PL = powerlifters; NC-PL = non-competitive powerlifters; EQ-PL = equipped powerlifters; ↓↑ = maintained; ↓ = decreased; ↑ = increased. ^†^ = indicates outcome and result post-overload week (i.e., increased volume-load similar to planned overreaching). Williams conducted a 3-week study where week 1 was normal training, week 2 was an overload week, and week 3 was a taper. Williams compared week 2 to week 3/week 1 to week 3 outcomes. Godawa et al. tapered intensity by −10% during the first 7 days of the taper followed by −15% during the final 7 days of the taper. When combing groups, Godawa et al. showed statistical significance for combined BS-1RM (*p* = 0.02), DL-1RM (*p* = 0.001), PT (*p* = 0.005), and Wilks score (*p* = 0.03) improvements. Andre, Askow et al. increased training intensity by 10% over week 1–3 of the taper (85% to 95% 1RM) then decreased intensity to 91% during week 4. * = denotes survey/qualitative studies. No performance outcome data were provided for qualitative studies.
